# Supplementary material for: Identification of CD73 as a Novel Biomarker Encompassing the Tumor Microenvironment, Prognosis, and Therapeutic Responses in Various Cancers
Source: Cancers (Basel). 2022 Nov 17;14(22):5663. doi: 10.3390/cancers14225663 (PMC9688912; doi:10.3390/cancers14225663)
Supplement: Supplementary file 1 [file cancers-14-05663-s001.zip › cancers-1973050-supplementary.pdf]

cancers MDPI

Supplementary Materials

Identification of CD73 as a Novel Biomarker Encompassing the Tumor Microenvironment, Prognosis, and Therapeutic Responses in Various Cancers

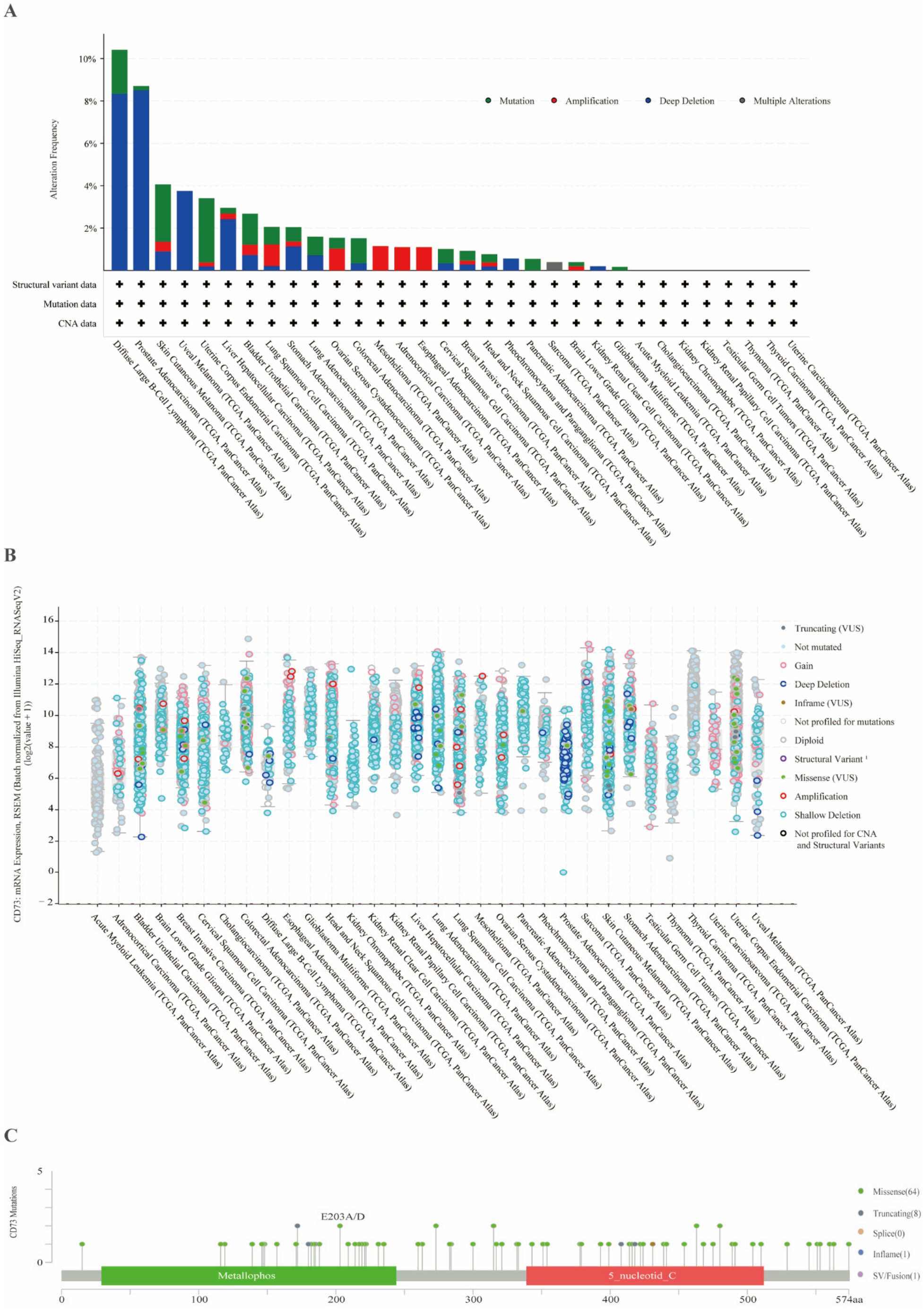

Figure S1. Mutation aspect of CD73 in pan-cancer. Alteration frequency of CD73 in pan-cancer (**A**). CD93 mRNA expression, mutation type, structural variant, and copy number based on the

cBioPortal analysis (**B**). Mutation profile of CD73 across protein domains (**C**). ACC: Adrenocortical carcinoma; BRCA: Breast invasive carcinoma; CESC: Cervical squamous cell carcinoma and endocervical adenocarcinoma; CHOL: Cholangiocarcinoma; COAD: Colon adenocarcinoma; ESCA: Esophageal carcinoma; GBM: Glioblastoma; HNSCC: Head and neck squamous cell carcinomas; KICH: Kidney chromophobe; KIRC: Kidney renal clear cell carcinoma; KIRP: Kidney renal papillary cell carcinoma; LAML: Acute myeloid leukemia; LGG: Low-grade glioma; LIHC: Liver hepatocellular carcinoma; LUSC: Lung squamous cell carcinoma; O.V.: Ovarian Cancer; PAAD: Pancreatic adenocarcinoma; PCPG: Pheochromocytoma, and paraganglioma; PRAD: Prostate adenocarcinoma; READ: Rectum adenocarcinoma; SARC: Sarcoma; SKCM: Skin cutaneous melanoma; STAD: Stomach adenocarcinoma; TGCT: Testicular germ cell tumors; THCA: Thyroid carcinoma; THYM: Thymoma; UCS: Uterine carcinosarcoma; UVM: Uveal melanoma; LSCC: laryngeal squamous cell carcinoma; UTUC: upper tract urothelial cancer; PSCC: Penile squamous cell carcinoma.

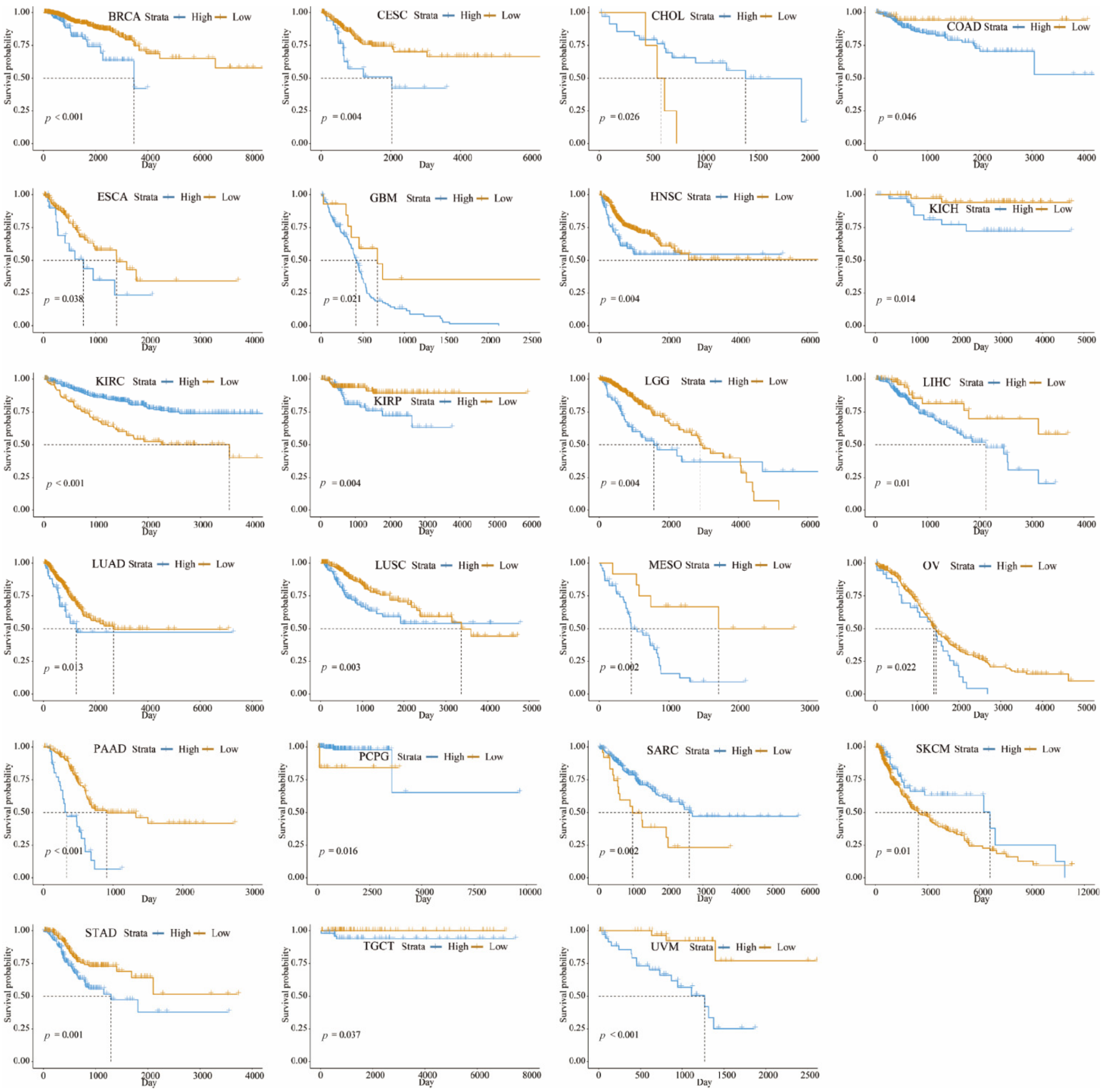

Figure S2. KM displayed the prognostic value of CD73 on DSS in pan-cancer.

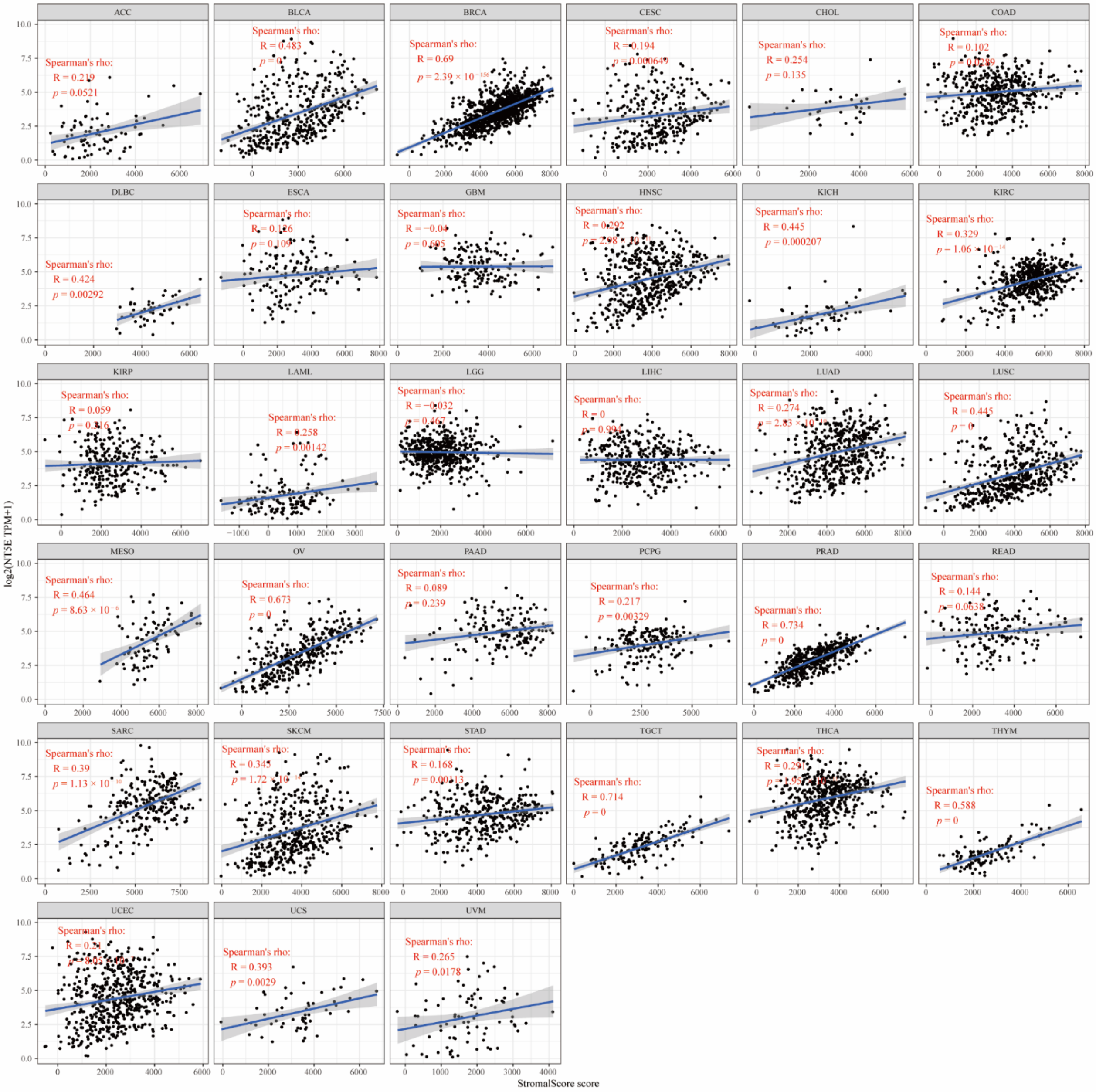

Figure S3. Relationship between CD73 expression and the stromal scores.

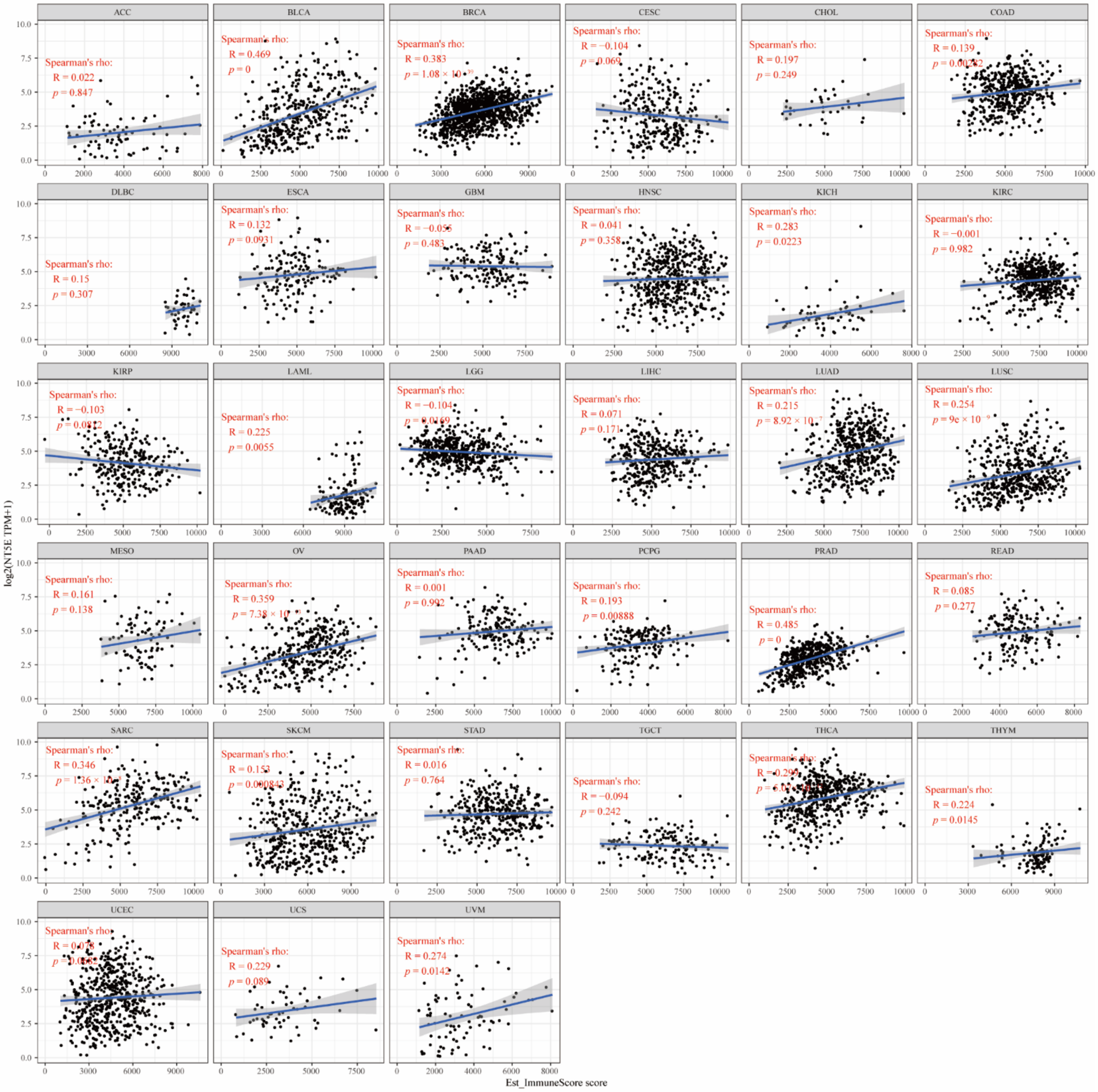

Figure S4. Relationship between CD73 expression and the immune scores.

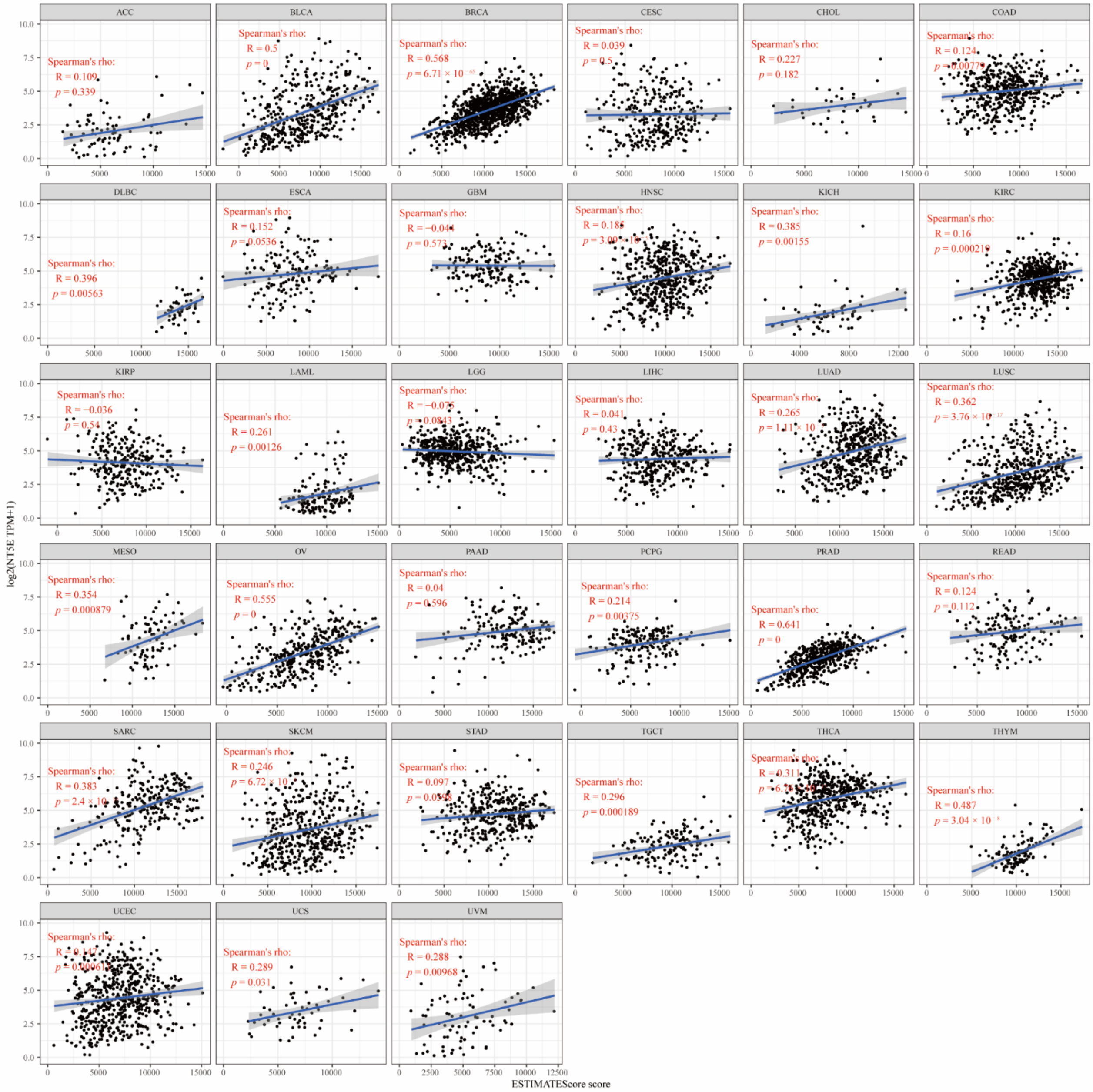

Figure S5. Relationship between CD73 expression and the estimate scores.

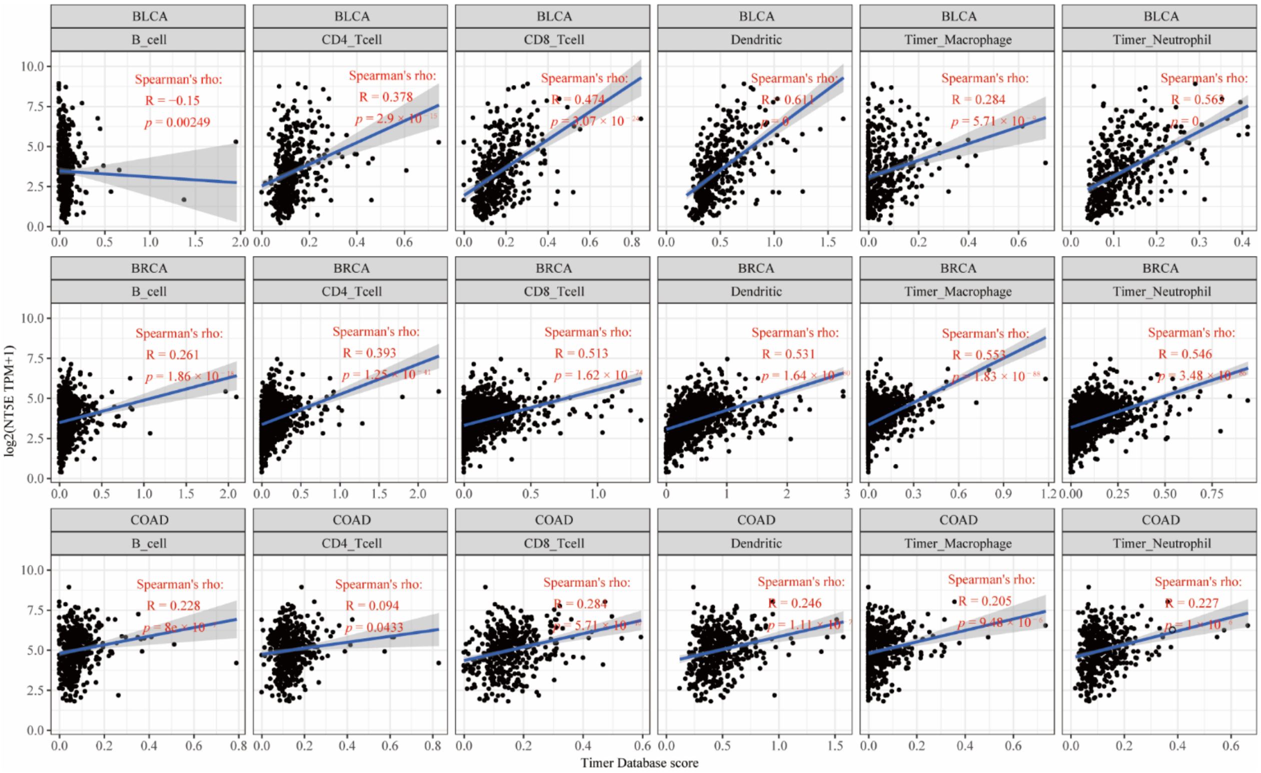

Figure S6. Top three cancers related to the infiltrating immune cells in the TME based on CD73 levels.

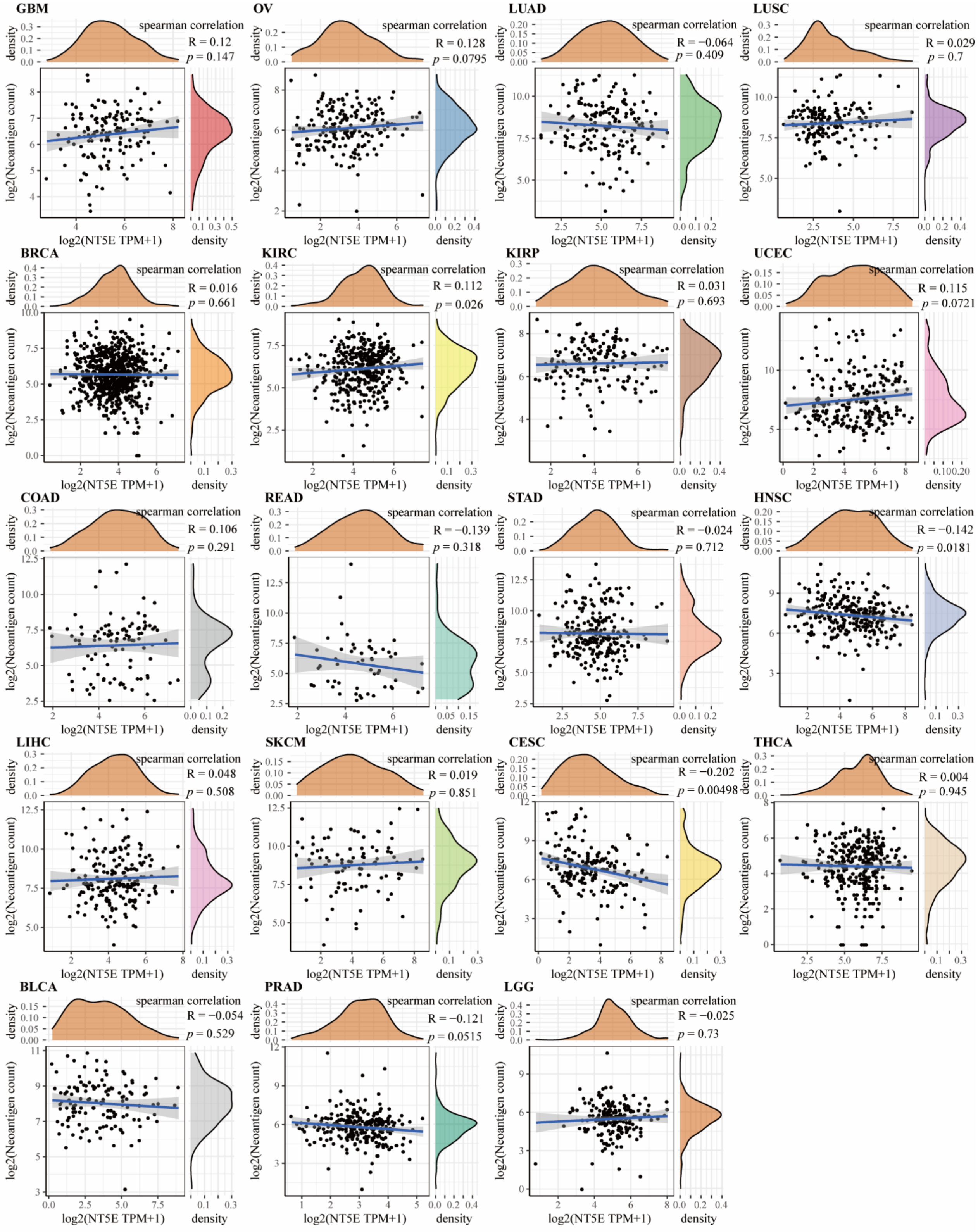

Figure S7. Relationship between CD73 levels and neoantigens in pan-cancer.

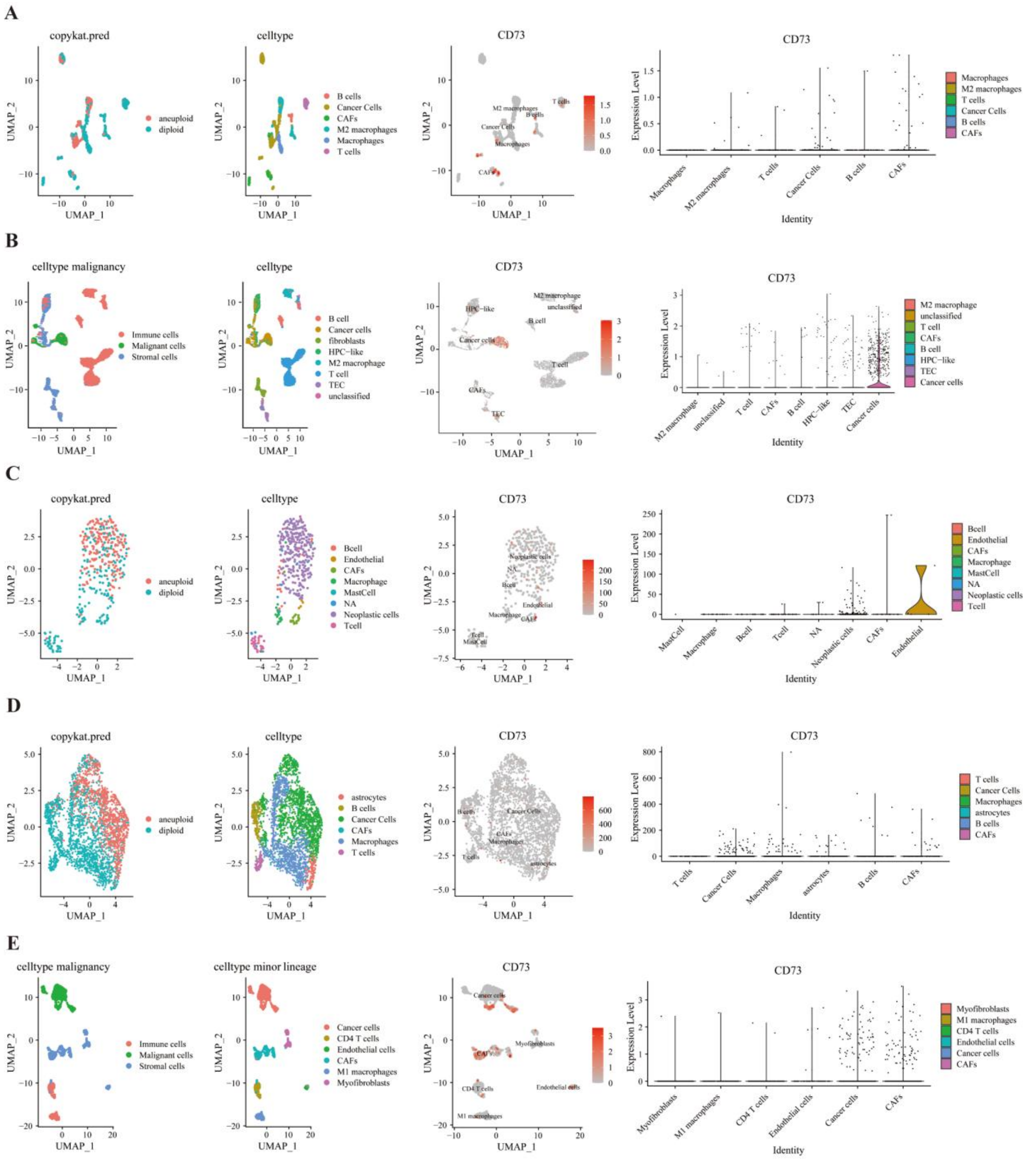

Figure S8. Single cell sequencing analysis CD73 on tumor and stromal cells in the TME: BRCA (**A**), CHOL (**B**), CRC (**C**), STAD (**D**), and OV (**E**).

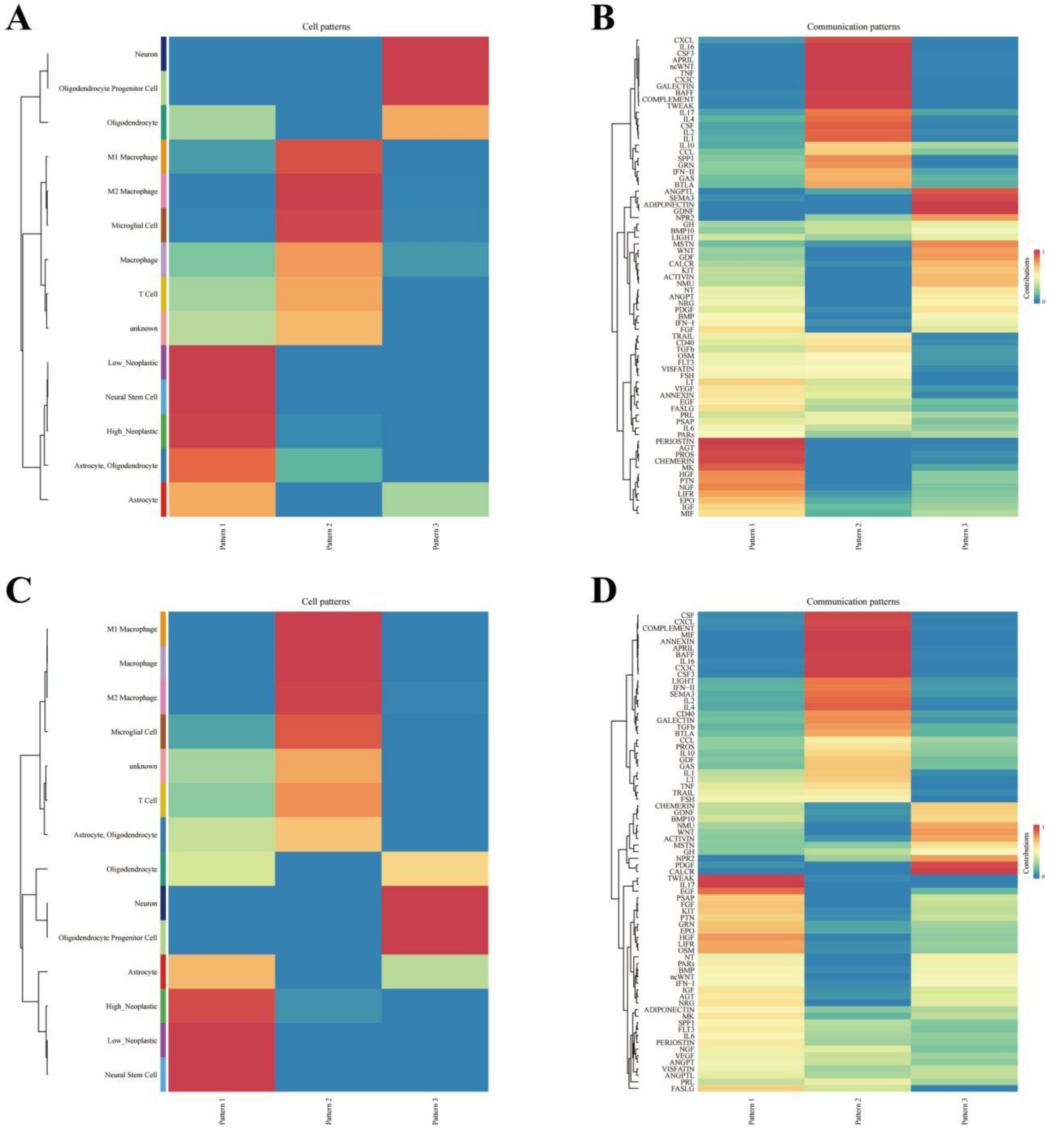

Figure S9. The cellular communication of the 14 cell types in GBM. The cell patterns and specific genes regarding the receiver of the 14 cell types (**A**,**B**). The cell patterns and specific genes regarding the sender of the 14 cell types (**C**,**D**).

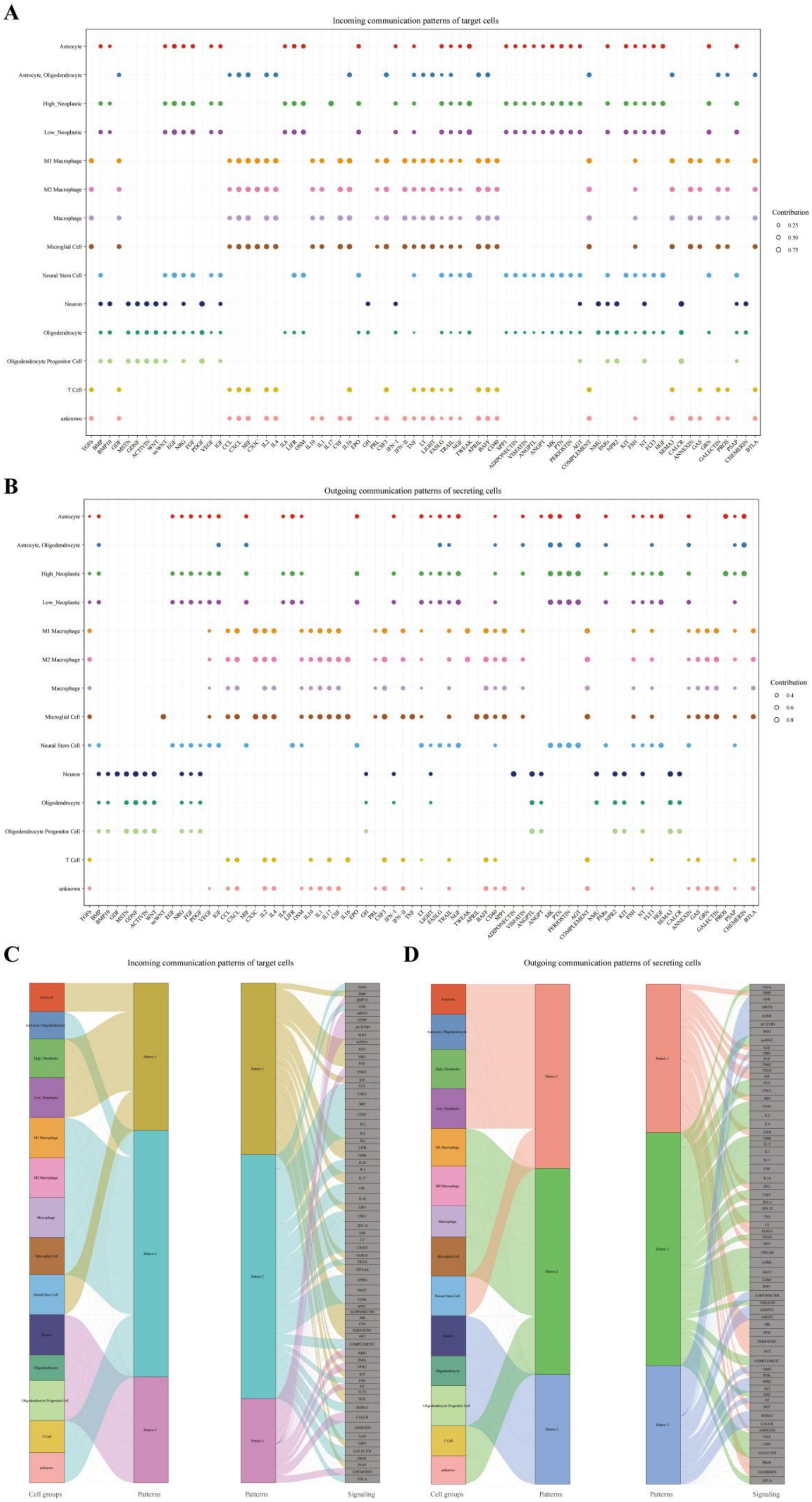

Figure S10. The communication patterns of the receiver and sender for 14 cell types. The dot plots visualize the receiver communication and sender communication patterns of the 14 cell types in GBM (**A**,**B**). The river plot depicts the receiver communication and sender communication patterns of the 14 cell types in GBM (**C**,**D**).

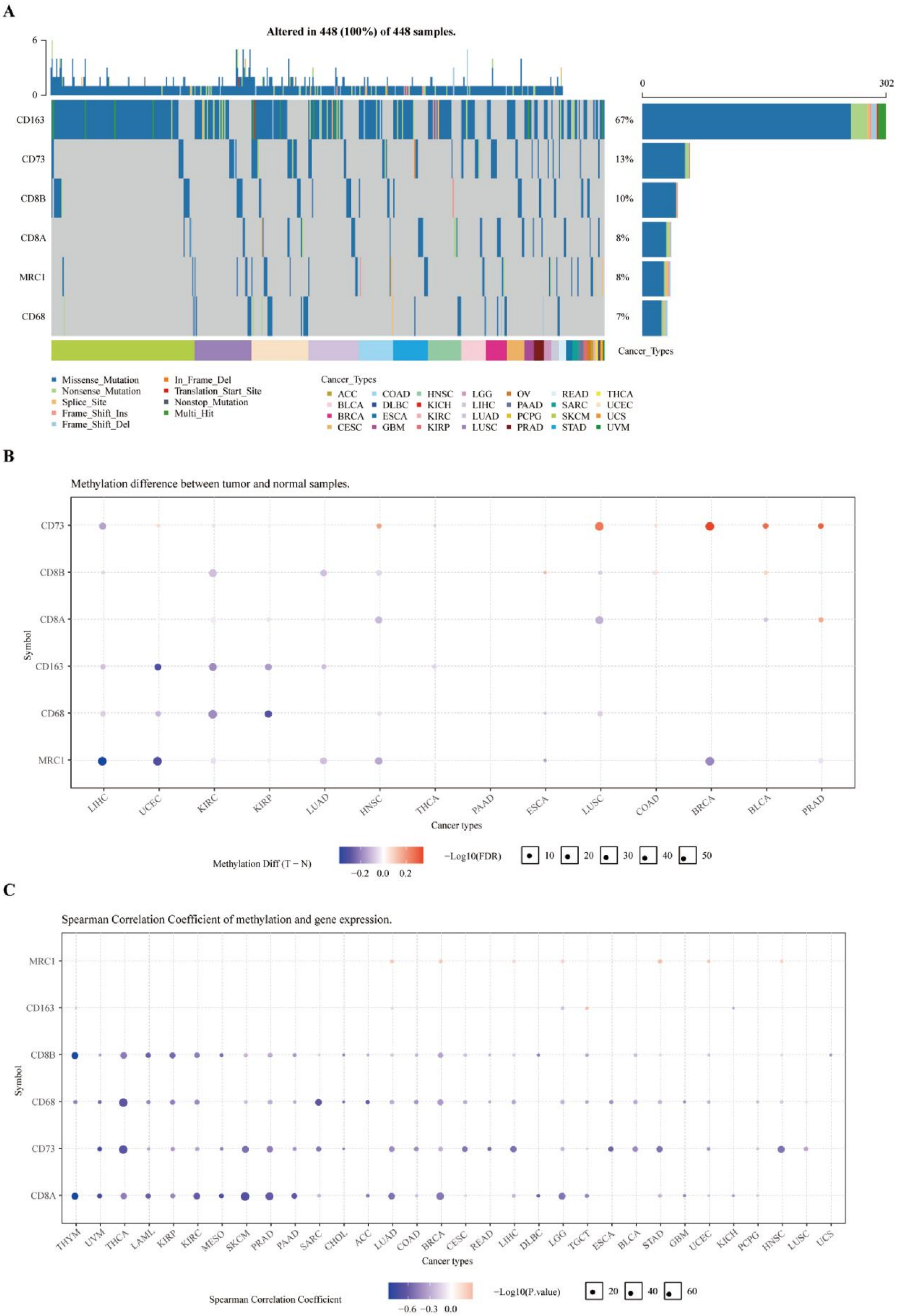

Figure S11. Mutational characteristics of CD73 and other markers from the GSCA website. Altered mutations of CD73 and markers of macrophages and T cells in pan-cancer (**A**). Methylation difference of CD73 and markers of macrophages and T cells in pan-cancer (**B**). Correlation between methylation and gene expression of CD73 and markers of macrophages and T cells in pan-cancer (**C**).

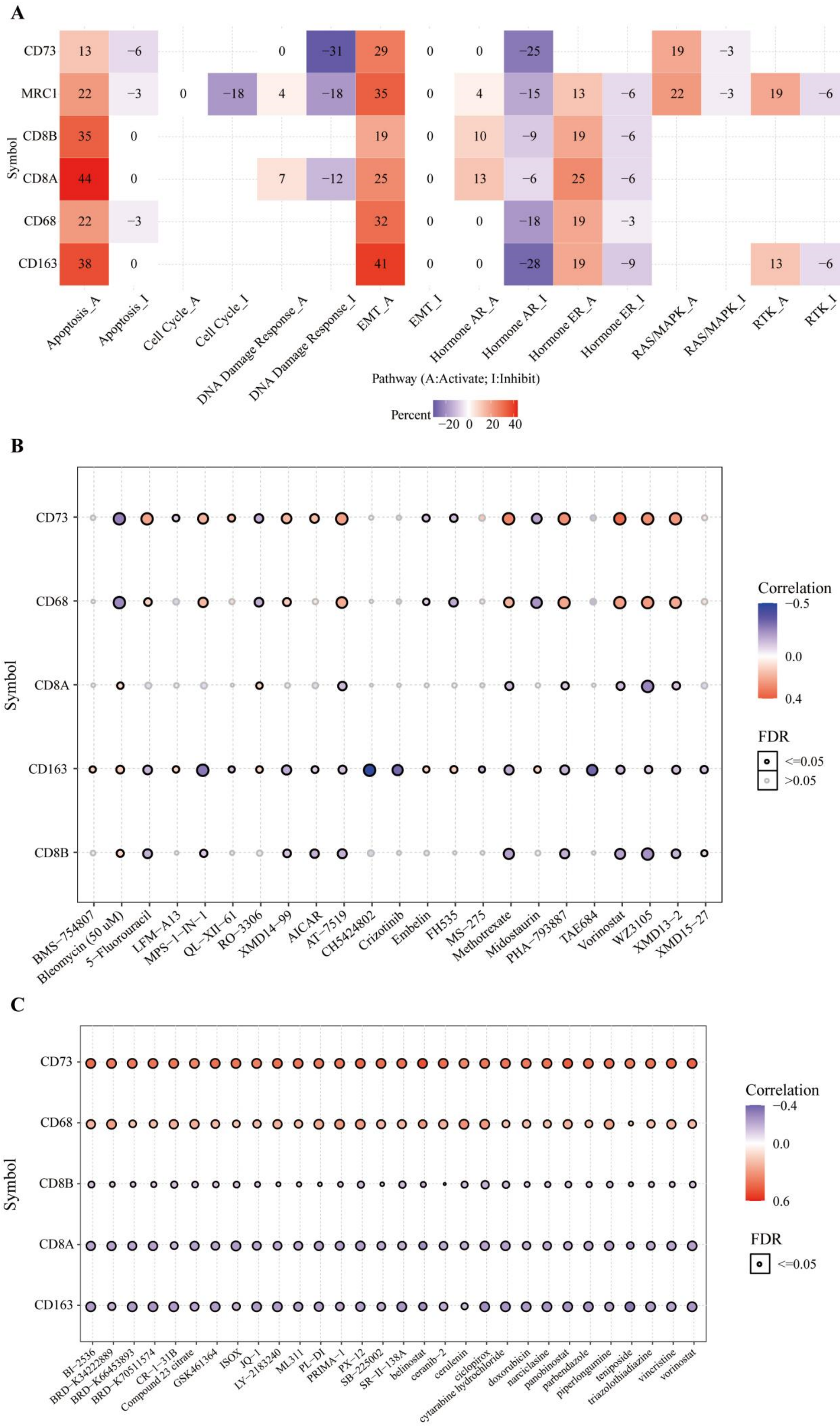

Figure S12. Enrichment analysis and drug prediction of CD73 and other markers from the GSCA website. Enrichment pathways of CD73 and other markers (**A**). Drug sensitivity prediction of CD73 and other markers based on the GDSC (**B**) and CTRP (**C**) databases.
